# The Clinical Features of FLAIR-Hyperintense Lesions in Anti-MOG Antibody Associated Cerebral Cortical Encephalitis with Seizures: Case Reports and Literature Review

**DOI:** 10.3389/fimmu.2021.582768

**Published:** 2021-06-11

**Authors:** Yun-feng Wang, Xue-wu Liu, Jian-ming Lin, Ji-ye Liang, Xiu-he Zhao, Sheng-jun Wang

**Affiliations:** ^1^ Department of Neurology, Qilu Hospital, Cheeloo College of Medicine, Shandong University, Jinan, China; ^2^ Department of Neurology, Yucheng People's Hospital, Dezhou, China

**Keywords:** myelin oligodendrocyte glycoprotein, encephalitis, seizure, cortical, fluid attenuated inversion recovery

## Abstract

**Background:**

The presence of fluid attenuated inversion recovery (FLAIR)-hyperintense lesions in anti-myelin oligodendrocyte glycoprotein (MOG) antibody-associated cerebral cortical encephalitis with seizures (FLAMCES) was recently reported. However, the clinical characteristics and outcome of this rare clinico-radiographic syndrome remain unclear.

**Methods:**

The present study reported two new cases. In addition, cases in the literature were systematically reviewed to investigate the clinical symptoms, magnetic resonance imaging (MRI) abnormalities, treatments and prognosis for this rare clinico-radiographic syndrome.

**Results:**

A total of 21 cases were identified during a literature review, with a mean patient age at onset of 26.8 years. The primary clinicopathological characteristics included seizures (100%), headache (71.4%), fever (52.3%) and other cortical symptoms associated with the encephalitis location (61.9%). The common seizure types were focal to bilateral tonic-clonic seizures (28.6%) and unknown-onset tonic-clonic seizures (38.1%). The cortical abnormalities on MRI FLAIR imaging were commonly located in the frontal (58.8%), parietal (70.6%) and temporal (64.7%) lobes. In addition, pleocytosis in the cerebrospinal fluid was reported in the majority of the patients (95.2%). All patients received a treatment regimen of corticosteroids and 9 patients received anti-epileptic drugs. Clinical improvement was achieved in all patients; however, one-third of the patients reported relapse following recovery from cortical encephalitis.

**Conclusions:**

FLAMCES is a rare phenotype of MOG-associated disease. Thus, the wider recognition of this rare syndrome may enable timely diagnosis and the development of suitable treatment regimens.

## Introduction

Myelin oligodendrocyte glycoprotein (MOG) is a membrane protein expressed on the surface of oligodendrocytes and in myelin sheaths (1). The primary clinical phenotypes for MOG-IgG-positive disorders are optic neuritis and myelitis (2, 3). Recent studies have suggested that MOG-associated demyelinating disease may be an entity distinct from multiple sclerosis (MS), acute disseminated encephalomyelitis (ADEM) and neuromyelitis optica spectrum disorder (NMOSD) (1, 3).

Epileptic seizures have been recorded in ~20% of patients with MOG-associated diseases (4). Furthermore, encephalitis accompanied by seizures and cortical lesions is a rare anti-MOG phenotype, which was first reported by Ogawa in 2017 (5). All 4 patients with MOG-IgG positivity described by Ogawa were observed to have experienced seizures and exhibited unilateral hyperintense cortical lesions on magnetic resonance imaging (MRI) fluid attenuated inversion recovery (FLAIR) sequences. Recently, Budhram systematically reviewed the literature and identified 20 similar cases with clinical symptoms (seizures, headache, fever and cortical symptoms); the MRI scan identified cortical FLAIR-hyperintense lesions in anti-MOG-associated encephalitis with seizures (FLAMES) (6). It is difficult to comprehend why Budhram performed investigations in some patients without seizure symptoms, despite naming this rare phenotype FLAMES (6). The present study hypothesized that the primary features of this MOG phenotype are the clinical manifestations of cortical encephalitis and seizures, as well as the radio-imaging changes in the cerebral cortical FLAIR hyperintense lesions on MRI. We named this phenotype “FLAMCES”, which stands for FLAIR-hyperintense lesions in anti-MOG antibody-associated cerebral cortical encephalitis with seizures.

The characteristics and outcome of the seizures of this rare syndrome have not been investigated to date. In the present study, two new similar cases were reported, and a systematic review of previous cases presenting with FLAMCES was conducted. The study aimed to further characterize the clinical features and outcome of this rare clinico-radiographic syndrome associated with anti-MOG antibodies.

## Materials and Methods

The literature was searched for reported cases of encephalitis with seizures and cortical lesions following MRI scans in the presence of anti-MOG antibodies. The repositories PubMed, Ovid, EBSCOhost and ScienceDirect were searched for the terms’ [encephalitis] AND [MOG]’, ‘[MOG] AND [seizure]’ and ‘[MOG] AND [cortical] AND [MRI]’. All relevant published articles from January 2017 to May 2020 were reviewed for potential study inclusion. Cases were included in the present study if they met the following criteria: i) presented with encephalitis accompanied by seizures; ii) exhibited distinctive unilateral/bilateral cortical FLAIR hyperintensity on MRI without the involvement of the adjacent juxta-cortical white matter; iii) the presence of MOG-IgG antibodies were identified by cell-based assay (CBA) in the serum; and iv) infectious encephalitis [e.g., herpes simplex virus (HSV), syphilis] and other autoimmune diseases (e.g., multiple sclerosis, NMOSD, Hashimoto’s encephalitis, rheumatological diseases, autoimmune encephalitis associated with synaptic receptors/neuronal cell surface proteins antibodies) were rationally excluded. Cases were excluded if insufficient data were provided in the literature. Discrepancies between reviewers regarding the inclusion of cases were resolved by discussion. Information of the enrolled cases and our cases are presented in the **Table 1**. Two new cases diagnosed in our hospital were also included in the present study, for which the patients provided written informed consent. The present study was approved by the Ethics Committee of Qilu Hospital of Shandong University.

**Table 1:** The clinical manifestations, diagnostic examination and outcome of FLAMCES.

	**Case1**	**Case2**	**Case3**	**Case4**	**Case5**	**Case6**	**Case7**	**Case8**	**Case9**	**Case10**	**Case11**	**Case12**	**Case13**	**Case14**	**Case15**	**Case16**	**Case17**	**Case18**	**Case19**	**Case20**	**Case21**
**Sex**	M	M	M	M	M	M	F	M	M	M	F	F	M	M	F	F	M	M	M	M	M
**Age(years)**	19	23	39	36	23	38	17	46	27	29	20	11	31	19	19	25	37	23	23	32	28
**Fever**	+	-	-	-	-	-	-	+	+	+	+	+	+	-	+	-	-	+	-	+	+
**Headache**	+	+	–	–	+	+	–	+	+	–	+	+	+	+	+	–	–	+	+	+	+
**Seizure types FBTCS FAS GTCS UTCS Unclassified**	+	+	+	+	+	+	+	+	+	+	+	+	+	+	+	+	+	+	+	+	+
**SE**	–	–	–	–	–	–	–	–	–	–	ND	ND	ND	ND	–	–	–	–	–	–	–
**Other symptoms Hemiparesis Aphasia Hemianopsia Vision loss Memory defect Psychiatry Paraplegia**	++	++	++	++	-	+++	++	+	++	+	-	+	-	+	+	++	++	++	+++	-	+
**Phenotypes before encephalitis/time duration (month)**	CE(84)	–	RON(7)	–	–	–	ADEM(76)	–	–	–	–	–	–	BiON	–	–	–	–	–	CE(60)	–
**Phenotypes after encephalitis/time duration (month**	-	-	-	-	-	-	ADEM(30)	RON(23)	-	-	RON; BSS	RON; LON	LON; BSS ;ADEM	ME	-	ON; ME	-	-	-	-	-
**Anti-MOG antibody Serum/CSF**	1:32/ND	1:10/1:1	1:512/1:32	1:2048/1:4	1:256/1:16	1:1024/-	1:1024/ND	ND	1:1024/ND	1:1024/ND	1:10/ND	1:10/ND	1:320/1:1	1:10/ND	1:256/1:128	ND	ND	ND	1:1000/ND	1:10/ND	1:10/ND
**CSF WC (/mm^3^) Lymphocyte%**	120 56	30 98	29 88	63 98	101 50	311 41	137 73	56 93	205 67	73 ND	15 ND	76 ND	142 ND	3 ND	200 72	67 80	120 71	599 34	105 69	100 ND	105 ND
**CSF protein (g/L)**	0.49	0.77	0.35	0.38	0.86	0.53	ND	0.36	0.84	ND	ND	ND	ND	ND	0.59	ND	ND	↑	ND	ND	ND
**CSF OB**	-	-	ND	-	-	-	+	+	ND	-	ND	ND	ND	ND	ND	+	-	-	-	ND	ND
**EEG slow waves**	RH	LH	RH	No	RH	ND	LH	No	LH	BF	ND	ND	ND	ND	RH	ND	ND	LH	ND	ND	ND
**MRI cortical FLAIR hyperintensity**	R Fr/P	L Fr/T/O/I	R Fr/P	R Fr/P	R P	L Fr/P/T/O/I	L Fr/P/T	Bi P	L Fr/P/T/O/I;R:Fr	Bi Fr/P	R Fr/P/T/I	R T	R P/T/O	R:P L:T	R Fr/P/T	L T	R Fr/P/T	L Fr/P	R T	L Fr/T/P	L T/I
**MRI meningeal enhancement**	–	+	ND	ND	ND	ND	ND	+	ND	+	ND	ND	ND	ND	+	–	–	+	+	–	+
**MRI other abnormality**	-	spinal cord	-	-	-	-	-	cingulate gyri	-	-	-	-	-	-	spinal cord	-	-	-	-	pontine	optic nerve
**Immunotherapy drugs and duration (month)**	MP	MP	MP(18)	MP(24)	DEX	MP	MP	MP	MP	MP	MP ;TAM	MP ;AZA	MP ;MMF	MP ;AZA	MP	MP ;RTM	MP	MP	MP	MP	MP
**Anti-epileptic drugs**	LVT	LVT	CBZ, LTG	CBZ	CBZ	CBZ	ND	ND	ND	ND	ND	ND	ND	ND	LVT	ND	ND	LVT	LVT	ND	ND
**Recovery after treatment**	+	+	+	+	+	+	+	+	+	+	+	+	+	+	+	+	+	+	+	+	+
**Seizures free after treatment**	+	+	+	+	+	+	+	+	+	+	+	+	+	+	+	+	+	+	+	+	+
**Follow-up duration (month)**	18	9	30	40	23	72	30	65	ND	ND	ND	ND	ND	ND	5	ND	ND	6	ND	8	ND
**Relapse times during follow**	0	0	0	0	0	0	1	1	0	0	2	2	3	2	0	2	0	0	0	0	0

MOG, myelin oligodendrocyte glycoprotein; M, male; F, female; N, no; Y, yes; ME, myelitis; ADEML, acute demyelinating encephalomyelitis like; CE, cerebral cortical encephalitis; R, right; L, left; Bi, bilateral; ON, optic neuritis; BSS, brainstem syndromes; MD, memory decline; FBTCS, focal to bilateral tonic-clonic seizure; FAS, focal aware motor onset seizure; GTCS, generalized tonic-clonic seizure; UTCS, unknown onset tonic-clonic seizure; SE, status epilepticus; RH, right hemisphere; LH, left hemisphere; BF, bilateral frontal lobes; Hp, hemiparesis; Ho, hemianopsia; ND, no data available/reported; CSF, cerebrospinal fluid; WC, white cell; Fr, frontal lobe; P, parietal lobe; T, temporal lobe; O, occipital lobe; I, insular lobe; DEX, dexamethasone; MP, methylprednisolone; RTM, rituximab; CBZ, carbamazepine; LTG, lamotrigine; LVT, levetiracetam; TAM, tacrolimus; AZA, azathioprine; MMF, mycophenolate mofetil; SW, slow waves; OB, oligoclonal band; FLAIR, fluid attenuated inversion recovery; MRI, magnetic resonance imaging; EEG, electroencephalography.

Case 3-6 from Ogawa (reference 5); Case 7 from Fukushima (reference 7); Case 8 from Fujimori (reference 8); Case 9 from Adachi (reference 9); Case 10 from Ikeda (reference 10); Case 11-14 from Wang (reference 11); Case 15 from Sugimoto (reference 12); Case 16-17 from Cobo-Calvo (reference 3); Case 18 from Budhram (reference 6); Case 19 from Haddad (reference 13); Case 20-21 from Tao (reference 14).

## Case Reports

### Case 1

**Figure 1:**
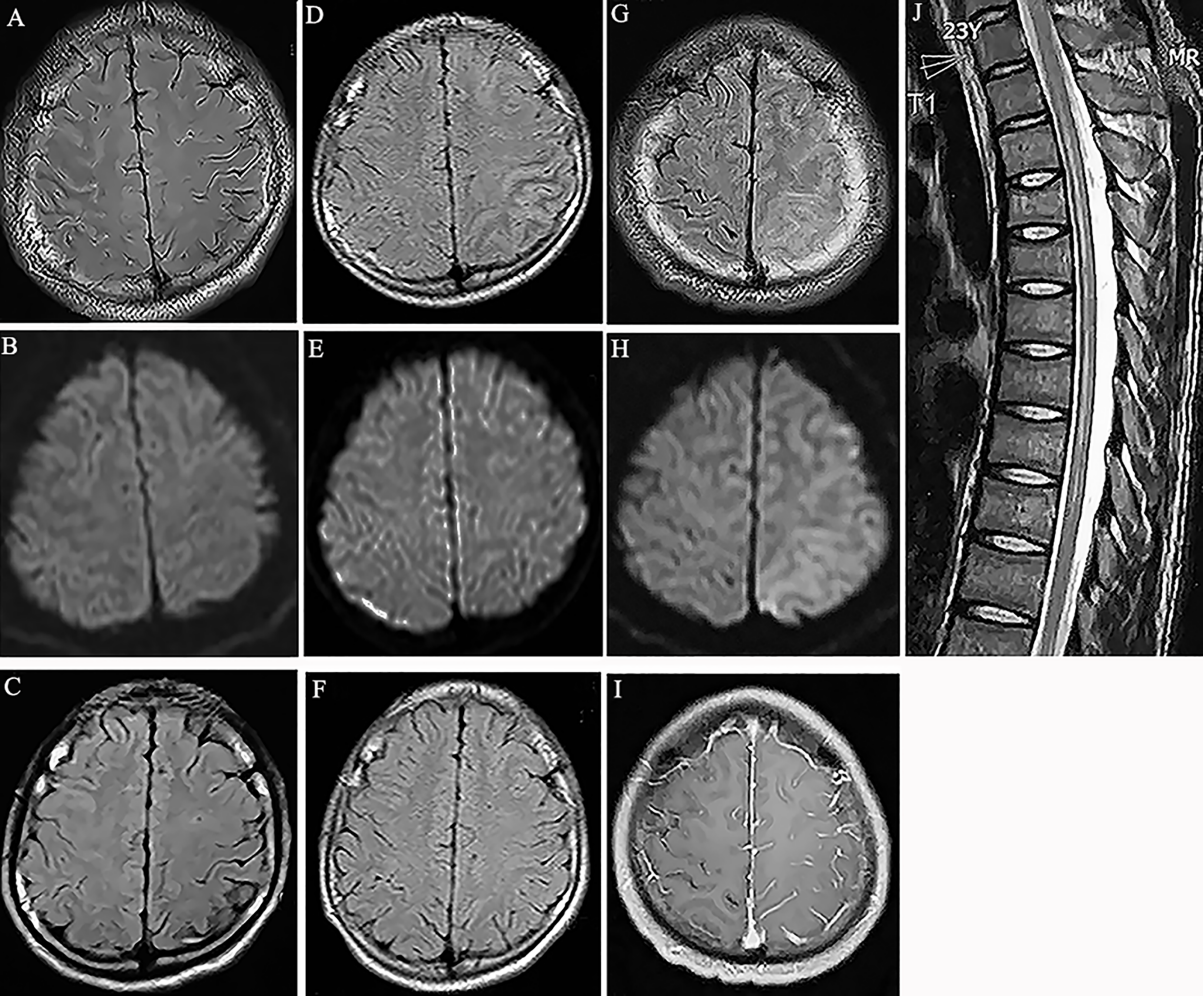
**(A)** Fluid attenuation inversion recovery (FLAIR) image showed hyperintensity in the cortical region of right frontal and parietal lobes without white matter involvement (Case 1). **(B)** Diffusion weighted imaging (DWI) showed no obvious signal change in the cortex (Case 1). **(C)** MRI scan showed the abnormality disappeared at the follow-up scan (Case 1). **(D)** Hyperintensity in the cortical regions of left parietal lobes in MRI FLAIR images 7 years before this episode (Case 1). **(E)** DWI showed no obvious signal change in the cortex (Case 1). **(F)** MRI scan showed the abnormal lesions disappeared at the follow-up scan (Case 1). **(G)** Hyperintensity in the cortical regions of left frontal and parietal lobes in MRI FLAIR images (Case 2). **(H)** DWI showed mild hyperintensity in the involved cortical regions (Case 2). **(I)** Gadolinium enhanced T1-weighted image showed meningeal linear enhancements in the sulci of left brain lobes (Case 2). **(J)** T2-weighted image showed hyperintensity in the spinal cord from thorax 2 to 9 segments. (Case 2).

A 19-year-old man was admitted to the hospital due to experiencing seizures and blurred vision, with prodromal headache and fever. The patient developed a focal motor seizure and a secondary generalized tonic-clonic seizure. His vision acuity of right and left eye was 3.9 and 4.8 respectively by logarithmic visual acuity chart examination. His left upper limb power was grade 4 (MRC). The brain MRI scan revealed obvious hyperintensity on the FLAIR image not diffusion weighted imaging (DWI) in the right frontal and parietal cortex (**Figures 1A, B**). Examination of the cerebrospinal fluid (CSF) revealed normal pressure, but increased pleocytosis (120/μl) and protein (0.49 g/l) levels. The screening of the CSF by RT-PCR for the presence of HSV, Epstein-Barr virus, cytomegalovirus and returned negative results. The syphilis infection was excluded by TPPA and RPR testing. The presence of serum anti-MOG antibodies was positive (titer 1:32), as determined using a CBA. The results for anti-aquaporin-4 (AQP4) antibody, anti-N-methyl-D-aspartate receptor antibody, anti-leucine-rich glioma inactivated 1 (LGI1) antibody, anti-contactin-associated protein-like 2 (CASPR2) antibody, α-amino-3-hydroxy-5-methyl-isoxazolepropionic acid receptor (AMPAR) antibody, gamma-aminobutyric acid (GABA) receptor, and other autoimmune antibodies, including antinuclear, anti-ds-DNA, anti-thyroid and anti-neutrophil cytoplastic antibodies, were all negative. Subsequently, oxcarbazepine (600mg per day) and intravenous methylprednisolone (500mg per day for 5 days) were administered, followed by a dose of oral prednisone (1 mg/kg per day), with the doses gradually decreasing over time. Mycophenolate mofetil was added with a dose of 1 gram per day. The neurological symptoms of the patient resolved following treatment. In fact, a brain MRI scan after 4 weeks revealed that the abnormal cortical hyperintensity had almost disappeared (**Figure 1C**).

The patient’s history revealed that he had been admitted to the hospital 7 years prior due to a seizure and headache. At the time, the brain MRI scan revealed hyperintensity in the left frontal and parietal cortex on the FLAIR images. In addition, a CSF examination revealed mildly elevated levels of white blood cells. Following treatment with glucocorticoids, the patient completely recovered and was diagnosed with steroid-responsive encephalitis of unknown etiology (**Figures 1D–F**).

### Case 2

A 23-year-old male patient presented with a headache, generalized tonic-clonic seizures and altered mental status, without a fever. In addition, no demyelination syndrome was reported. Two days later, the patient presented with weakness in both lower limbs and a brain MRI scan revealed the presence of diffuse cortical lesions in the left cerebral hemisphere (**Figures 1G–I**). The cognitive test score of this patient was 20 by mini-mental state examination (MMSE). His right nasolabial fold became shallow. His right and left upper limb power were grade 3 and grade 5, respectively. His right and left lower limb power were grade 2 and grade 3, respectively. He showed active bilateral knee tendon reflexes with positive bilateral Babinski sign. The spinal cord MRI scan identified the presence of myelitis from the T2 to the T9 segments (**Figure 1J**). In addition, the CSF examination reported 30 lymphocytes/μl and elevated protein levels of 0.77 g/l. Extensive screening by RT-PCR, TPPA, RPR and immunofluorescence testing for the presence of infections, including HSV, Epstein-Barr virus, cytomegalovirus and syphilis, and for the presence of autoimmune antibodies associated with synaptic receptors/neuronal cell surface proteins in the CSF, returned negative results. The serum antinuclear, anti-SS-A, anti-SS-B, anti-ds-DNA, anti-thyroid and anti-neutrophil cytoplastic antibodies were all negative. The anti-AQP4 antibody was also negative; however, the serum anti-MOG antibody was positive (titer 1:32). The patient was treated with intravenous methylprednisolone (500mg per day for 5 days), mycophenolate mofetil (1 g per day) and levetiracetam (1.5g per day). Then oral prednisone gradual tapered from a dose of 60 mg per day. Three weeks later, the patient had recovered fully and no longer experienced seizures. The glucocorticoid dosage was gradually reduced, and no relapse was reported over the following 9 months.

## Results

An additional 19 cases from 11 different literature reports were identified and included in the review in addition to our two new reported patients (2, 5–14).

The mean age at onset of the patients was 26.8 years (range, 11-46 years) and 76.2% of the patients were male. The type of seizure experienced included focal aware motor onset (1/21), focal to bilateral tonic-clonic (6/21), unknown onset tonic-clonic (8/21) and unclassified (6/21)-seizures (due to inadequate information or inability to place in other categories). No patients developed status epilepticus. The frequency of the other symptoms reported among these cases was as follows: 15/21 (71.4%) headache, 11/21 (52.3%) fever and 13/21 (61.9%) cortical symptoms, including aphasia, hemiparesis (8/21), hemianopsia (4/21), memory defect (4/21) and psychiatric symptoms (5/21). In addition, 7/21 patients presented with other clinical phenotypes, including optic neuritis (5/21) and myelitis (2/21), whereas 11/21 patients experienced relapse (range, 1-3 episodes) before or/and after the encephalitis episode. The previous phenotype before the onset of the encephalitis phenotype included optic neuritis (2/21), ADEM-like syndrome (1/21) and encephalitis (2/21). During the follow-up, 7 patients reported relapses, including optic neuritis (5/21), myelitis (2/21), brainstem syndrome (2/20) and ADEM-like syndrome (2/21) following the manifestation of the encephalitis phenotype (**Table 1**).

All patients presented with cortical hyperintensity on the MRI FLAIR sequence, without involvement of the white matter. In addition, 17/21 patients presented with unilateral cortical abnormalities, including in the frontal (10/17), parietal (12/17), temporal (11/17), insular (4/17) and occipital (3/17) lobes, and 4/21 patients exhibited bilateral abnormalities within the frontal (3/4), parietal (3/4), temporal (2/4), insular (1/4) and occipital (1/4) lobes. Brain MRI enhancement data were reported in 11 cases, and 7/11 patients presented with meningeal enhancement. Lesions in the spinal cord (2/21), brainstem (1/21) and optic nerves (1/21) were also reported on MRI. Pleocytosis in the CSF was observed in 20/21 patients. Furthermore, EEG data were available for 11 cases and slow waves were reported in 9/11 patients. The number of white blood cells in the CSF ranged from 3- to 599/ml and the protein levels ranged from 0.35- to 0.84 g/l. A total of 12 patients underwent a CSF oligoclonal band screen and 3 cases were found to be positive. The titer of the MOG antibody in the serum ranged from 1:10 to 1:2,048. In addition, 6 patients were found to have MOG antibodies in the CSF, with a titer ranging from 1:1 to 1:128 (**Table 1**).

All patients received treatment with corticosteroids, such as methylprednisolone (20/21) and dexamethasone (1/21). Anti-epileptic drugs, including levetiracetam (5/9), carbamazepine (4/9) and lamotrigine (1/9), were also administered as a single agent or in combination in 9 patients. Immunosuppressive drugs, including azathioprine (2/5), rituximab (1/5), tacrolimus (1/5) and mycophenolate mofetil (1/5) were also administered to 5 patients presenting with relapse. Both clinical and radiographic recovery was achieved in all patients following therapy, and no unprovoked seizures were reported in any of the patients following treatment. However, one-third of the patients (7/21) reported relapse during the follow-up period (**Table 1**).

## Discussion

In the present case report and literature review, the features of a rare phenotype of FLAMCES were characterized. In addition, the study reported two novel cases of this rare phenotype of MOG-associated disease. There were some unique clinical features of our patients compared with what had reported in literature. One patient experienced two attacks with clinical features of FLAMCES. The other one had clinical features with both FLAMCES and myelitis during one attack.

MOG-antibody-associated disease phenotypes are a new entity in the spectrum of inflammatory demyelinating diseases (1, 3). The most frequent clinical presentations include optic neuritis, myelitis, acute disseminated encephalomyelitis-like syndrome and brainstem syndrome (3). Less typical syndromes include encephalitis with seizures, cranial nerve involvement and chronic lymphocytic inflammation with pontine perivascular enhancement, which is responsive to steroids (3). Patients with MOG antibody-associated disease are more likely to manifest with encephalitis-like symptoms characterized by seizures compared with those with AQP4-IgG-associated disease (15). In one cohort study, nearly one fifth of MOG-antibody-positive patients presented with encephalitis, while unique cortical lesions were observed in 72.2% of the patients (11). In these cases, the majority of the patients with encephalitis were reported to have white matter lesions on the MRI scan. A total of 5 patients also overlapped with anti-N-methyl-D-aspartate-receptor encephalitis. Thus, only 4 patients fit the criteria of the present study. Ogawa first described 4 adult MOG antibody-positive cases presenting with unique unilateral encephalitis with epileptic seizures (5). All the cases enrolled in the present study presented with seizures and, amongst these, the focal to bilateral tonic-clonic seizure type was commonly observed. Since the data describing the characteristics of the seizures were not complete for the majority of the cases, the seizure types of these patients were classified as unknown onset tonic-clonic seizure or unclassified seizure. Furthermore, status epilepticus was not reported in any of these patients, which is a common occurrence in autoimmune encephalitis associated with antibodies against neuronal cell surface proteins. Consistent with the findings of Budhram, the present study reported the presence of headaches and fever in the majority of the patients (6). Cortical symptoms associated with the encephalitis lesions, including aphasia, hemiparesis, hemianopsia, memory impairment and psychiatric symptoms, were also reported in several cases. In addition, approximately half of the patients suffered an optic neuritis episode. Optic neuritis in the setting of the encephalitic episode or following an episode is a highly probable event (2).

MRI findings in MOG encephalomyelitis usually include an ADEM-like pattern with diffuse signal changes in the cortical grey matter, juxtacortical/subcortical/deep white matter and deep grey matter, according to FLAIR images (11, 15). The distinctive brain MRI findings in patients associated with MOG antibodies are thalamic and pontine lesions, while cortical lesions are only found in 16.3% of the cases (2, 3). Zhong reported that seizures in MOG-associated disease are commonly associated with cortical and subcortical brain lesions (16). Recently, Armangue also reported that, in several pediatric patients with non-ADEM encephalitis with positive MOG antibodies, 18% involved cortical or cortical-subcortical lesions. The majority of these pediatric patients presented with cognitive impairment, epilepsy and generalized cortical atrophy during the follow-up (17). In addition, Ogawa initially described the rare MOG clinico-radiographic syndrome as exhibiting distinctive characteristics, i.e., unilateral cortical lesions without the involvement of white matter in MRI scans. Budhram reported that several patients with bilateral cortical involvement demonstrated a broader disease spectrum (6). In the present study, ~20% patients in the literature reported bilateral cortical abnormalities on MRI FLAIR images. The most common sites of cerebral cortical lesions were identified as the frontal, temporal and parietal lobes. The cortical lesions on MRI FLAIR images should be carefully differentially diagnosed from HSV encephalitis, Creutzfeldt-Jakob disease, metabolic encephalopathy (hypoglycemia, hyperammonemia or hypoxemia) or mitochondrial encephalomyopathy.

The most interictal EEG findings are non-specific slow waves consistent with the location of the cortical lesions. CSF pleocytosis was also reported in almost all cases. In addition, the white blood cell count in the CSF is usually elevated with lymphocytic predominance in patients positive for MOG antibodies (18). Pathological examination has identified lymphocytic infiltration in both the subarachnoid space and brain parenchyma without distinct demyelinating plaques and neuronal/axonal loss (8, 10). Experimental studies have also established MOG-IgG as a pathogenic antigen rather than a secondary immune reaction (19). The present study discovered that the titer of MOG antibodies in the serum widely varied among the patients, and was not associated with disease severity or outcome. The concomitant release of autoimmune antibodies detected in the same patient with an autoimmune disease is not a rare clinical phenomenon. For example, encephalitis associated with MOG antibodies may overlap with N-methyl-Daspartate-receptor (NMDAR) antibodies (20, 21); and Jarius reported that concomitant autoimmune disorders were present in 8% of MOG-IgG-positive patients (18).

The syndrome of cerebral cortical encephalitis with seizures associated with MOG antibodies was initially described as a steroid-responsive disease (2). The acute seizures are likely triggered by an episode of demyelination, and immunotherapy was discovered to be more useful compared with anti-epileptic drugs (AEDs) for controlling the seizures (2). The present study also discovered that the seizures of several patients were completely resolved by steroids without any AEDs. AEDs, such as carbamazepine, lamotrigine and levetiracetam, were reported to be used to control the seizures. However, it remains difficult to accurately evaluate the efficacy of these AEDs alone in controlling seizures compared with immunotherapy.

The majority of patients with autoimmune antibody-mediated encephalitis develop seizures provoked by immune mechanisms; however, only a few patients develop epilepsy following recovery from encephalitis (4). The occurrence of seizures was completely abolished following recovery of encephalitis in the present literature review; no unprovoked epileptic seizures were reported following recovery from encephalitis. Thus, we prefer to name this rare MOG phenotype “cerebral cortical encephalitis with seizures” instead of “cerebral cortical encephalitis with epilepsy” (5, 14). Since MOG patients with an encephalitis phenotype experienced a sustained seizure-free period following immunotherapy, the long-term use of AEDs may be unnecessary (4, 15, 16). It remained unclear whether cortical lesions in patients were entirely inflammatory or some involve cortical demyelination. Pathologically, there was inflammatory changes in the cortex and subcortex with microglial proliferation in perivascular regions and subcortical white matter, which might suggest ‘preactive’ lesions of demyelination (10).

Although the rapid response to steroids is a distinctive feature in patients with MOG antibodies-associated disease, there is also a tendency for relapse following steroid withdrawal (3). Hamid described 5 cases of patients with MOG antibodies suffering from seizures and encephalopathy, and unprovoked seizures occurred in 2 of these patients. These cases exhibited involvement of the brain cortex, alongside hemispheric white matter and basal ganglia abnormalities on the MRI scan (15). This indicated that patients with abnormalities extending beyond the cortex on MRI scans have a high rate of seizure recurrence. Armangue reported that several patients with pediatric encephalitis-like MOG with extensive cortical involvement presented with severe brain atrophy, refractory epilepsy and cognitive changes (17). In several encephalitis cases, catastrophic brain injury and death occurred due to intracranial hypertension (7, 17). Thus, the phenotype of encephalitis with seizures should not be simply considered as a benign syndrome.

In conclusion, FLAMCES is a rare clinico-radiographic syndrome of MOG antibody-associated disease, whereby tonic-clonic seizures are common. FLAMCES may combine with myelitis or optic neuritis during one attack and relapse. Following treatment with glucocorticoids, patients may completely recover from encephalitis and cortical lesions, as demonstrated by MRI imaging. However, AEDs should not be used as long-term treatment and long-term immunosuppression should be considered instead for patients with relapse.

## Data Availability Statement

The raw data supporting the conclusions of this article will be made available by the authors, without undue reservation.

## Ethics Statement

The studies involving human participants were reviewed and approved by The Ethics Committee of Qilu Hospital of Shandong University. The patients/participants provided their written informed consent to participate in this study. Written informed consent was obtained from the individual(s) for the publication of any potentially identifiable images or data included in this article.

## Author Contributions

Y-fW, X-wL, J-mL and J-yL collected the clinical data and reviewed literature. X-hZ and S-jW analyzed the data. Y-fW, X-wL and S-jW wrote the manuscript. All authors contributed to the article and approved the submitted version.

## Funding

This work is supported by grants from Innovative Research Project of Resident Standardization Training of Qilu Hospital, Shandong University (No.ZPZX2019A04); Undergraduate Teaching Reform and Research Project of Cheeloo College of Medicine, Shandong University (No.qlyxjy-201917), The Quality Improvement Plan for Postgraduate Education in Shandong Province (No.26010182037203), and The Taishan Scholars Program of Shandong Province.

## Conflict of Interest

The authors declare that the research was conducted in the absence of any commercial or financial relationships that could be construed as a potential conflict of interest.
